# 

**Published:** 1893-06

**Authors:** 


### The Library of the Bristol Medico-Chirurgical Society.

### PERIODICALS REQUIRED TO COMPLETE SETS.

*Any of the following addressed to* L. M. Griffiths, *Honorary Librarian of the Bristol Medico-Chirurgical Society, 28 Berkeley Square, Bristol, will be gratefully acknowledged:*

Alienist and Neurologist, The. All before 1891.

American Gynecological Society, Transactions of the. All before and after 1891

American Journal of Obstetrics, The. All before 1884 and between 1885 & 1892.

American Journal of the Medical Sciences, The. All before 1889 and Vols. XCIX. and C.

American Practitioner and News, The. All before 1886.

American Surgical Association, Transactions of the. Vols. III.—VIII.

Annals of Surgery. All before 1889 and Vol. XI.

Archives de Neurologie. All before 1892.

Archiv für pathologische Anatomie und Physiologie und für klinische Medicin. All before and after 1861.

Army—Statistical Reports on the Sickness, &c., among the Troops in the United Kingdom, &c. All before and after 1853.

Army Medical Department—Statistical, Sanitary and Medical Reports. All before 1862; between 1864 and 1890; and after 1890.

Asclepiad, The. All before 1891.

Association of American Physicians, Transactions of the. Vol. I. and all after Vol. III.

Australasian Medical Directory. All before 1892.

Australasian Medical Gazette, The. All before 1889.

Australian Medical Journal. All before 1888 and Vol. XI.

Bibliotheca Medico-Chirurgica. All before 1889.

Birmingham Medical Review, The. All before Vol. XXV. and Vol. XXVI.

Brain. All before and after Vol. XIII.

Bristol Health Reports. All before 1891.

British Gynæcological Journal, The. Vols. I. and IV.

British Medical Journal. Vol. I. for 1890.

Bulletin de l'Académie de Médicine. All before 1891.

Bulletin de l'Académie Royale de Belgique. All before 1891.

Calendar of the Royal College of Surgeons. All before 1869; 1870-73; 1879-90

Canada Medical and Surgical Journal. All before and after Vol. XVI.

China Medical Missionary Journal, The. Vol. I.

Cincinnati Lancet and Clinic. All before Vol. LII., Vols. LIII.-LXII., LXVI., LXVII.

Clinical Society's Transactions. All before and after Vol. X.

Collective Investigation Record, The. All after Vol. II.

Dentists Register. All before and after 1885.

Deutsche medicinische Wochenschrift. All before 1891.

Dublin Journal of Medical Science, The. Vols. I.–LII., LVIII.–LXI., LXIII.–LXXI., LXXIII., LXXIV., LXXVI., LXXXI.–LXXXVII.

Edinburgh Medical Journal. Vols. I., VIII., X., XI., XIV.–XVI., XXII.–XXXIV.
Epidemiological Society, Transactions of the. All before 1875. All between 1880 and 1883. 1884. All after 1889.

Gazette de Gynécologie. Tomes I.–V.
Gazette des Hôpitaux. All before 1891.
Glasgow Medical Journal, The. Vols. I.–XX., XXXIII.

Hospitals—
Guy's Hospital Reports. Series I. and II.
Johns Hopkins Hospital Reports. Vol. I. and Nos. 1–4 of Vol. II.
Middlesex Hospital. Registrars' Reports. All before 1889.
University College Hospital. Reports. All before 1886; 1887; all after 1888

Illustrirte Monatsschrift der ärztlichen Polytechnik. All before 1890.
Index Medicus. Vols. I.–X.
Indian Medical Gazette. Vols. I.–XXV.
International Medical Congress, Transactions of the. All before and after 1881

Jahrbücher, Schmidt's. Bd. 1–96, 105–108, 113, 114, 123–126, and all after the year 1866.
Journal of Anatomy and Physiology. Vols. I.–X., XIII.–XIX., and all after Vol. XXI.
Journal of Cutaneous and Venereal Diseases. Vols. I.–III.
Journal of Laryngology and Rhinology. Vols. I.–IV.
Journal of Microscopy. Vols. I.–X.
Journal of Nervous and Mental Disease, The. Vols. I.–XV.
Journal of the American Medical Association, The. Vols. I.–XIV.

Laboratory Reports, Royal College of Physicians, Edinburgh. Vol. I.
Lancet, The. All before 1825; 1826–27; Vol. II. for 1828; 1829–32; 1845–48; 1850–56; 1861–64; 1866; 1870–75; 1879–81; 1884–90.
Liverpool Medico-Chirurgical Journal, The. Vols. I.–VII., X.
Local Government Board, Annual Reports of the. All before 1871; between 1871 and 1886; after 1887.
London Medical Record, The. Vols. I.–XI.
London Medical Recorder, The. All after 1889.
London Medical Review. All except Vol. I.
London Medical and Surgical Journal, The. All after Vol. VII.
London, Transactions of the Pathological Society of. Vols. I.–III. and all after XLII.
Lunacy, Reports of the Commissioners in. All before 1850 and after 1852.

Marine-Hospital Service of the United States, Annual Report of the Supervising Surgeon-General of the. All before 1889 and after 1891.
Medical Age, The. Vols. I.–VI.
Medical Annual, The. All before 1887.
Medical Bulletin, The. Vols. I.–XIII.
Medical Circular. All before and after Vol. IV.
Medical Council, Minutes of the General. All before and after 1887.
Medical Directory, The. All before 1870; 1871–73; 1876.
Medical News, The. Vols. I.–LVIII., LX.–LXV.
Medical Officer of Privy Council, Reports of. First five Reports, seventh and eighth Reports, and all after ninth Report.
Medical Record. Vols. I.–XXIII.
Medical Register. All except 1868, 1871, and 1885.

Medical Review. Vols. I.-XXIV.
Medical and Surgical Reporter, The. Vols. I.-LV., LVII.-LXI.
Medical Temperance Journal, The. Vols. II.-VII., and all after Vol. XV.
Medical Times, The. Vols. I.—XI., XVI.
Medical Times and Gazette, The. Vol. II. for 1876; 1877; 1879-80; all after 1885.
Medicinische Bibliographie. All before 1888.
Medico-Chirurgical Review, The. All after Vol. IV.
Medico-Chirurgical Transactions. Vol. XII., Part II.; XIII., Part I.; XIV., Part II.; XVI., Part II.-XVIII.; all after 1889.
Monthly Journal of Medical Science, The. Vols. I.-VIII. and all after Vol. XX.
Montreal Medical Journal. Vols. I.-XVII.

Navy—Statistical Reports on the Health of the Navy. All before 1854; 1855-88; 1890; all after 1891.
New York Medical Journal, The. Vol. I.-XXXVII.
New Zealand Medical Journal, The. Vols. I., II.

Obstetrical Transactions. Vol. XIII. and all after XXVI.

Practitioner, The. All after Vol. IV.
Prisons, Report of the Directors of Convict. All except 1864.
Progrès médical, Le. Tomes I.-VI.
Provincial Medical and Surgical Journal. Vol. I. 1840-41.

Registrar-General of Births, Deaths, and Marriages in England, Reports of. All before 1846; 1847-55; 1863-64; 1866-86.
Répertoire général d'Anatomie et de Physiologie pathologiques, et de Clinique chirurgicale. All after Tome VI.
Retrospect of Medicine, Braithwaite's. Vols. III., LII., LXXVII., LXXX., LXXXIV., LXXXVI.-CIV.
Revue de Laryngologie, d'Otologie et de Rhinologie. Tomes I.-X.
Revue médicale, La. Tome I. and all after Tome II.
Royal Academy of Medicine in Ireland, Transactions of the. Vols. I.-VII.

Sanitarian, The. Vols. I.-XVII., XXI., and all after XXIII.
Sanitary Institute of Great Britain, Transactions of the. All after Vol. XI.
Sanitary Record, The. All before and after 1886.
Sei-I-Kwai Medical Journal, The. Vols. I.-VII.
Sperimentale, Lo. All before 1889 and after 1891.
St. Andrew's Medical Graduates' Association, Transactions of the. All after Vol. VI.
St. Louis Medical and Surgical Journal, The. Vol. I.-LVI., LVIII., LIX.
Surgeons in London, List of the Members of the Royal College of. All before 1825 and after 1827.
Surgeons in England, List of the Fellows and Members of the Royal College of. All before and after 1844.

Therapeutic Gazette, The. Vols. I.-VII.

Union médicale, L'. All before 1891.

West London Medico-Chirurgical Society, Transactions of the. Vol. I. and all after Vol. II.
Würzburger medicinische Zeitschrift. All before 1861 and after 1863,

Year-Book of Scientific and Learned Societies. All before 1890.

Year-Book of Treatment. All before 1884; 1887-88; 1890.

Zeitschrift der k. k. Gesellschaft der Arzte in Wien. All before 1862 and after 1864.

### THE FOLLOWING VOLUMES ARE ALSO NEEDED TO COMPLETE SETS:

**Andral, G.** ... ... *Clinique médicale.* All after Tome V.
**Arnott, N.** ... ... *Elements of Physics.* 5th Ed. Vol. II.

**Bell, J. and C.** ... *Anatomy and Physiology of the Human Body.* 5th Ed. Vol. I.
**Bennett, J. H.** ... *Text-Book of Physiology.* Part I.
**Bourneville, Dr.** ... *Recherches sur l'Epilepsie, l'Hystérie, et l'Idiotie.* Vol. I.

**Casper, J. L.** ... *Forensic Medicine.* New Sydenham Society. Vol. IV.
**Charcot, J. M.** ... *Œuvres complètes.* All except Tome V.
*Clinical Lectures, German.* New Sydenham Society. First Series.

**Dupuytren, Baron** . *Leçons Orales de Clinique Chirurgicale.* All after Tome II.

**Ewald, C. A.** ... ... *Lectures on Diseases of the Digestive Organs.* New Sydenham Society. Vol. I.

**Fyfe, A.** ... ... ... *A Compendium of Anatomy.* 4th Ed. Vol. II. 5th Ed. Vol. I.

**Good, J. M.** ... ... *Study of Medicine.* 3rd Ed. Vol. V.
**Gregory, G.** ... ... *Practice of Physic.* Vol. I. 1823.
**Griffin, J. J.** ... ... *Chemical Recreations.* 8th Ed. All except Part I.

**Hallerus, A.** ... .. *Disputationes ad Morborum Historiam et Curationem facientes.* Vol. VII.
**Henoch, E.** ... ... *Lectures on Children's Diseases.* New Sydenham Society. Vol. I.
**Home, Sir E.** ... *Lectures on Comparative Anatomy.* Vols. I. and IV.

**Klebs, E.** ... ... *Die Allgemeine Pathologie.* All except Zweiter Theil.
**Klein, E.** ... ... *Anatomy of the Lymphatic System.* Part II.

**Marshall, J.** ... ... *The Human Body.* Plates.
*Miscellanea Curiosa Medico-physica.* All except Vols. IV.–VII.
**Müller, J.** ... ... *Elements of Physiology.* Part I.

**Parry, C. H.** ... ... *Elements of Pathology and Therapeutics.* 2nd Ed. Vol. II.
**Paxton, J.** ... ... *Introduction to the Study of Human Anatomy.* Vol. II.
**Pott, P.** ... ... ... *Chirurgical Works.* Vol. I. 1783.
**Prichard, J. C.** ... *Researches into the Physical History of Mankind.* 3rd Ed. Vol. II.

**Santos, R. D.** ... *Clinique obstétricale.* All except Tome I.
**Sigmond** ... ... ... *On the Teeth and Gums.* All except Part I.

**Todd and Bowman** . *Physiological Anatomy.* 1st Ed. Vol. II. Parts I., II., IV.
**Trousseau, A.** ... *Clinical Medicine.* New Sydenham Society. Vol. V.

### The Library of the Bristol Medico-Chirurgical Society.

---

**On and after July 1st the Rooms in the Medical Wing of University College, Bristol, which are to be devoted to Library purposes, will be open to Members of the Society.**

---

*The following donations have been received since the publication of the list in March:*

*May* 31*st*, 1893.

| | |
|---|---|
| W. M. Barclay (1) ... ... ... ... ... ... ... | 3 volumes. |
| Alexander Carr (2) ... ... ... ... ... ... ... | 1 volume. |
| Mrs. J. P. Challacombe (3) ... ... ... ... ... | 124 volumes. |
| F. R. Cross, M.B. (4) ... ... ... ... ... ... | 2 ,, |
| J. K. Morgan (5) ... ... ... ... ... ... ... | 1 volume. |
| Bertram M. H. Rogers, M.D. (6) ... ... ... ... | 2 volumes. |
| Sir James Sawyer, M.D. (7) ... ... ... ... ... | 1 volume. |
| R. Shingleton Smith, M.D. (8) ... ... ... ... | 1 ,, |
| The Royal Medical and Chirurgical Society (9)... | 6 volumes. |

Unbound periodicals have been received from Mrs. Challacombe, Dr. Michell Clarke, and Mr. F. R. Cross.

### EIGHTH LIST OF BOOKS.

The figures in brackets refer to the figures after the names of the donors, and show by whom the volumes were presented. The books to which no such figures are attached have either been bought from the Library Fund or received through the *Journal*.

---

| | | | |
|---|---|---|---|
| **Aitchison, R. S.** ... | *A Medical Handbook* ... ... ... ... ... ... ... ... | | 1893 |
| **Ashwell, S.** ... ... | *Diseases of Women* ... ... ... ... ... | (3) 2nd Ed. | 1846 |
| **Averill, C.** ... ... | *Operative Surgery* ... ... ... ... ... ... | (3) 2nd Ed. | 1825 |
| **Badham, C.** ... ... | *On the Bronchial Inflammation* .. ... ... ... ... | (3) | 1808 |
| **Beausoleil, R.** ... | *Revue Statistique des Maladies de la Gorge, du Larynx, du Nez et des Oreilles* ... ... . ... ... ... | | 1893 |

| | | |
|---|---|---|
| **Bell, C.** ... ... ... | *The Anatomy of the Brain* ... ... ... ... ... ... (3) | 1802 |
| ,, ... ... ... | *Operative Surgery.* 2 vols.... ... ... ... (3) | 1807-9 |
| ,, ... ... ... | *On Injuries of the Spine and of the Thigh-bone* .. (3) | 1824 |
| **Bell, J.** ... ... ... | *The Anatomy of the Human Body.* 2 vols. (3) 2nd Ed. | 1797 |
| **Bennett, W. H.** ... | *Abdominal Hernia* ... ... ... ... ... ... ... | 1893 |
| **Blake, A.** ... ... | *Aphorisms illustrating Cases of Accouchement, &c.* (3) | 1817 |
| **Bramwell, B.**... ... | *Atlas of Clinical Medicine.* Vol. II., Part II. ... | 1893 |
| **Brodie, Sir B. C.** ... | *On Diseases of the Joints* ... ... ... (3) 4th Ed. | 1836 |
| **Browning, W. W.**... | *Modern Homeopathy* ... ... ... ... ... ... ... | 1893 |
| **Burns, J.** ... ... | *The Principles of Midwifery* ... ... (3) 5th Ed. | 1820 |
| **Celsus, A. C.** ... ... | *Medicinæ Libri Octo.* (Ed. by G. Futvoye) ... (3) | 1837 |
| ,, ... ... | *do.* (Tr. by J. Greive). (3) 3rd Ed. | 1838 |
| **Chamberlaine, W.**... | *Tyrocinium Medicum* ... ... ... ... (3) 2nd Ed. | 1819 |
| **Cheselden, W.** ... | *The Anatomy of the Human Body* ... (3) 13th Ed. | 1792 |
| **Clarke, Sir C. M.** ... | *On the Diseases of Females.* 2 parts. (3) 3rd Ed. | 1831 |
| **Cloquet, J.** ... ... | *Manuel d'Anatomie descriptive du Corps Humain.* ... ... ... ... ... ... ... ... 3 vols. (1) | 1825 |
| **Cooper, Sir A.** ... | *Lectures on the Principles and Practice of Surgery* (3) 2nd Ed. | 1830 |
| **Cowan, C.** ... .. | *A Bedside Manual of Physical Diagnosis* (3) 2nd Ed. | 1842 |
| **Cruveilhier, J.** ... | *Descriptive Anatomy.* (Tr. by W. H. Madden). 2 vols. ... ... ... ... ... ... ... (3) | 1841-42 |
| **Dewees, W. P.** ... | *Midwifery* ... ... ... ... ... ... ... ... ... (3) | 1825 |
| **Doyen, E.** ... ... | *Traitement Chirurgicale des Affections Inflammatoires et Néoplasiques de l'Utérus et de ses Annexes*... ... | 1893 |
| **Falconer, M.** ... ... | *Synopsis of Lectures on Anatomy and Surgery* ... (3) | 1777 |
| **Fyfe, A.** ... ... ... | *A Compendium of Anatomy.* 4th Ed. (3) Vols. I., III. | 1810 |
| **Gibson and W. Russell, G. A.** | *Physical Diagnosis.* ... ... ... 2nd Ed. | 1893 |
| **Gill, J. B.** ... ... | *An Epitome of Surgery* ... ... ... ... ... (3) | 1860 |
| **Graham, T. J.** ... | *The Best Methods of Improving Health.* (3) 6th Ed. | 1851 |
| **Gray, S. F.** ... ... | *A Supplement to the Pharmacopœias* ... ... ... (3) | 1818 |
| **Green, J. H.** ... ... | *The Dissector's Manual* ... ... ... ... ... (3) | 1820 |
| **Gregory, J.** ... ... | *On the Duties and Qualifications of a Physician* (3) 2nd Ed. | 1805 |
| ,, ... ... | *Conspectus Medicinæ Theoreticæ* ... (3) Edito Sexta | 1818 |
| **Griffin, J. J.** ... ... | *Chemical Recreations.* Part I. ... ... (3) 8th Ed. | 1838 |
| **Hamilton, A.**... ... | *On Female Complaints* ... ... ... ... ... ... (3) | 1792 |
| **Hamilton, J.** ... ... | *Management of Infants and Children* ... (3) 3rd Ed. | 1821 |
| **Harris, J. D.** ... ... | *Scab Healing*... ... ... ... ... ... ... ... ... | 1893 |
| **Harvey, W.** ... ... | *Anatomical Exercises* ... ... ... ... ... ... (3) | 1673 |
| **Hooper, R.** ... ... | *The Anatomist's Vade-Mecum* ... ... ... ... (3) | 1811 |
| | *Examinations in Anatomy, Physiology, &c.* (3) 3rd Ed. | 1814 |
| *Hospital-Ward to Consulting-Room, From* | ... ... ... ... ... ... ... | 1893 |
| **Hudson, T. J.** ... | *The Law of Psychic Phenomena* ... ... ... ... ... | [1892] |

Inman, T. ... ... *Foundation for a New Theory and Practice of Medicine* ... ... ... ... ... ... ... ... (3) 1860

Jones and E. H. Sieveking, C. H. *Pathological Anatomy* ... ... ... (3) 1854
Jones, C. W. ... ... *Surgical Lectures* ... ... ... ... ... ... ... ... (3) 1819

Kenwood, H. R. ... *Public Health Laboratory Work* ... ... ... ... ... 1893
Krafft-Ebing, R. v. *Psychopathia Sexualis.* (Tr. by C. G. Chaddock)... 1893

Laennec, R. T. H. .. *Treatise on Diseases of the Chest.* (Tr. by J. Forbes) (3) 4th Ed. 1827

Lankester, E. ... *On Food...* ... ... ... ... ... ... ... (3) 1864

" [Ed.] *Haydn's Dictionary of Popular Medicine* ... (3) 1874

Lescher, F. H. ... *The Elements of Pharmacy.* ... ... (3) 3rd Ed. 1869

Lewers, A. H. N. ... *Diseases of Women.* ... ... ... ... ... 4th Ed. 1893

Liston, R. ... ... *Practical Surgery* ... ... ... ... ... ... (3) 4th Ed. 1846

Mackenzie, C. ... *One Thousand Experiments in Chemistry* ... ... (3) 1821

Macready, J. F. C. H. *A Treatise on Ruptures* ... ... ... ... ... ... ... 1893

Mann, J. D. ... ... *Forensic Medicine and Toxicology* ... .. ... ... 1893

Marshall, J. ... ... *The Human Body.* Vol. I. ... ... ... ... (3) [1860]

Martinet, L. ... ... *Pathology.* (Tr. by J. Quain) ... ... (3) 2nd Ed. 1827

Merriman, S.... ... *Difficult Parturition* ... ... ... ... (3) 2nd Ed. 1814

*Mother's Thorough Resource Book, The* ... ... ... ... ... ... ... (3) [N.D.]

Nelson, J. ... ... *The Government of Children* ... ... ... ... (3) 1753

Odell, W. ... ... *Recreation* ... ... ... ... ... ... ... ... ... ... 1893

Paris, J. A. ... ... *Medical Chemistry* ... ... ... ... ... ... ... (3) 1825

Pepper, W. [Ed.]... *An American Text-Book of the Theory and Practice of Medicine.* Vol. I. ... ... ... ... ... ... ... ... 1893

*Pharmacopœia, British* ... ... ... ... ... ... ... ... ... ... ... (3) 1867

Phillips, J. ... ... *Outlines of the Diseases of Women* ... ... ... ... 1893

Pott, P. ... .. ... *Chirurgical Works.* Vols. II., III. ... ... ... (3) 1783

*Primitive Physick* ... ... ... ... ... ... ... ... ... ... (3) 2nd Ed. [1747]

Rayer, P. ... ... *Diseases of the Skin—Atlas.* (Tr. by R. Willis) (3) 1835

Reichenbach, Baron C. v. *Dynamics of Magnetism, Electricity, &c.* (3) 2nd Ed. 1851

Reynolds, J. R. .. *Diagnosis of Diseases of the Brain, &c.* ... ... (3) 1855

Ridge, J. J. ... ... *Alcohol and Public Health.* ... ... ... 2nd Ed. 1893

Russell, G. A. Gibson and W. *Physical Diagnosis.* ... ... ... 2nd Ed. 1893

Ryan, M. ... ... *The Medico-Chirurgical Pharmacopœia* (3) 2nd Ed. 1838

Sammarthanus, S.... *Pædotrophia.* (Tr. by H. W. Tytler) ... ... (3) 1797

Sawyer, Sir J. ... *Practical Medicine.* ... ... ... ... ... (7) 2nd Ed. 1891

Schuster, —.... ... *When may Syphilitics Marry?* (Ed. and Tr. by C. Renner) ... ... ... ... ... ... ... ... 1893

Shoemaker, J. V. ... *Materia Medica and Therapeutics.* 2 vols. ... ... 1893

Sieveking, C. H. Jones and E. H. *Pathological Anatomy* ... ... ... (3) 1854

Sigmond, —. ... ... *On the Teeth and Gums.* Part I. . . ... ... (3) 1825

Spurzheim, J. G. ... *Physiognomical System* ... ... ... ... (3) 2nd Ed. 1815

| | | | |
|---|---|---|---|
| Squire, B. | *On Rhinophyma* | (3) | 1889 |
| ,, | *Superfluous Hair* | | 1893 |
| Swayne, J. G. | *Obstetric Aphorisms.* | 10th Ed. | 1893 |
| Symonds, J. A. | *Waste* | (5) | 1863 |
| Tait, L. | *Diseases of the Ovaries* | (3) | 1874 |
| Tanner, T. H. | *Signs and Diseases of Pregnancy* | (3) | 1860 |
| Thomson, A. T. | *The London Dispensatory* | (3) 2nd Ed. | 1818 |
| Titley, J. M. | *Diseases of the Genitals of the Male* | (3) | 1829 |
| Walker, R. | *Memoirs of Medicine* | (3) | 1799 |
| Whitlock, R. | *Zootomia* | (3) | 1654 |
| Wilson, E. | *Diseases of the Skin* | (3) 2nd Ed. | 1847 |
| Ziemssen, O. | *Constitutional Syphilis* | | 1893 |

### TRANSACTIONS, REPORTS, JOURNALS, &c.

*The List of current Periodicals to be seen in the Library is given in the March number.*

Abstract of the Medical Sciences, Half-yearly. (3) Vol. XXX., 1860; (3) Vol. LVIII. 1873

American Practitioner and News, The ... ... Vols. XIII. and XIV. 1892

Archives cliniques de Bordeaux ... ... Tome I. 1892

Army Medical Department—Report for 1888 ... ... (9) Vol. XXX. 1890

Asclepiad, The ... ... Vol. IX. 1892

Australasian Medical Gazette, The ... ... Vol. XI. 1892

Australian Medical Journal, The ... ... Vol. XIV. 1892

British Medical Journal (3) Vol. I., 1869—Vol. I., 1879; (3) Vol. I. 1881
Bulletin de l'Académie Royale de Médecine de Belgique. Tome VI. 1892
Bulletin de l'Académie de Médecine. Tomes XXVII. & XXVIII. 1892

Cincinnati Lancet-Clinic, The ... ... Vol. LXVII. 1892

Clinical Journal, The ... ... Vol. I. 1893

Hospital, The ... ... Vol. XIII. 1892
Hospitals—
Johns Hopkins Hospital Reports. (9) Vol. II., Nos. 5, 6, 1891; Vol. III., Nos. 4, 5, 6 1893
University College Hospital Reports ... ... (6) 1886; (6) 1888
Hygiene and Demography, Transactions of the 7th International Congress of. 13 vols. ... ... 1892

Index Medicus ... ... Vol. XIV. 1892

Indian Medical Gazette, The ... ... Vol. XXVII. 1892

Journal of Anatomy and Physiology, The ... ... (4) Vol. XIV. 1879
Journal of Physiology, The ... ... ... ... ... ... ... (8) Vol. XII. 1891

Laboratory Reports. Royal College of Physicians, Edinburgh. (9) Vol. II. 1890
Leeward Islands Medical Journal ... ... ... ... ... ... Vol. II. 1892
Liverpool Medico-Chirurgical Journal, The ... ... ... Vol. XII. 1892
Local Government Board, Fifteenth and Sixteenth Annual Reports of the, 1885-86 and 1886-87 ... ... ... ... ... ... ... (9) 1886-87

Medical and Surgical Reporter, The... ... ... ... ... Vol. LXVI. 1892

Medical Bulletin, The ... ... ... ... ... ... ... ... Vol. XIV. 1892

Medical Chronicle, The ... ... ... ... ... ... ... Vol. XVII. 1893

Medical News, The ... ... ... ... ... ... ... ... Vol. LXI. 1892

Medical Press and Circular, The ... ... .. ... ... Vol. CV. 1892

Medical Register, The ... ... ... ... ... ... ... ... ... . . (2) 1868

Medical Temperance Journal, The. (3) Vol. I., 1870; (3) Vols. VIII.—XV. 1877-84

Medicinische Bibliographie ... ... ... ... ... ... ... ... ... 1892

New York Academy of Medicine, Transactions of the. 2nd Series. Vol. VIII. 1892
New Zealand Medical Journal, The ... ... ... ... ... ... Vol. V. 1892

Pathological Society of London, Transactions of the. (4) Vol. XLII. 1891
Progrès Médical, Le ... ... ... ... ... ... ... ... Tome XVI. 1892

Retrospect of Medicine, Braithwaite's (3) Vol. XL., 1860; (3) Vols. XLIX.—LXIV., 1864-72; (3) Vols. LXVI.—LXVIII. ... ... ... 1873-74

St. Louis Medical and Surgical Journal, The... ... ... Vol. LXIII. 1892

Therapeutic Gazette, The ... ... ... ... ... ... ... Vol. XVI. 1892

Year-Book of Scientific and Learned Societies ... ... ... ... ... 1893

### *PUBLICATIONS RECEIVED.*

From Allen & Hanburys, London:
*Clinical Charts.*

From Bureaux des *Archives provinciales de Chirurgie*, Paris:
*Traitement Chirurgical des Affections inflammatoires et Néoplasiqués de l'Utérus et de ses Annexes.* Par E. Doyen. 1893.

From John Barker & Co., London:
*Seventh Annual Report of Holloway Sanatorium.* 1892.

From "Christian Herald" Office, London:
15 *Predicted Events, from* 1893 *until the end of this Age on April* 11, 1901.

From J. & A. Churchill, London:
*The Leeward Islands Medical Journal.* Vol. II. 1892.
*Syphilis and the Nervous System.* By W. R. Gowers, M.D. 1892.
*Obstetric Aphorisms.* By Joseph Griffiths Swayne, M.D. 10th Edition. 1893.
*Superfluous Hair.* By Balmanno Squire, M.B. 1893.

From W. H. & L. Collingridge, London.
*Old Jonathan.* May, 1893.

From T. & A. Constable, Edinburgh:
*Atlas of Clinical Medicine.* By Byrom Bramwell, M.D. Vol. II., Part II. 1893.

From The F. A. Davis Co,, Philadelphia:
*Materia Medica and Therapeutics.* By John V. Shoemaker, M.D. 2nd Edition. 2 Vols. 1893.
*Psychopathia Sexualis.* By Dr. R. von Krafft-Ebing. 7th Edition. Translated by C. G. Chaddock, M.D. 1893.

From Samuel G. Dixon, M.D., Philadelphia:

*Involution Form of the Tubercle Bacillus and the Effect of Subcutaneous Injections of Organic Substances on Inflammations.* By Samuel G. Dixon, M.D. [Reprint.]

From Wm. J. Dornan, Philadelphia:
*Modern Homeopathy.* By William W. Browning, M.D. 1893.

From Feret et Fils, Bordeaux:
*Revue Statistique des Maladies de la Gorge, du Larynx, du Nez et des Oreilles.* Par Le Docteur R. Beausoleil. 1893.

From CHARLES GRIFFIN & COMPANY, LIMITED, London:
*A Treatise on Ruptures.* By JONATHAN F. C. H. MACREADY. 1893.
*Forensic Medicine and Toxicology.* By J. DIXON MANN, M.D. 1893.
*Outlines of the Diseases of Women.* By JOHN PHILLIPS, M.D. 1893.
*A Medical Handbook.* By R. S. AITCHISON, M.B. 1893.

From J. W. HATCH, Enfield:
*The Medical Pioneer.* April—June, 1893.

From Dr. H. H. HOWE, Vermont:
*The Abstract and Index.* December, 1892.

From THE JOHNS HOPKINS PRESS, Baltimore:
*The Johns Hopkins Hospital Reports.* Volume III., Nos. 4, 5, 6. 1893.

From JOURNAL PUBLISHING CO., Baltimore.
*Clinical Study and Analysis of 1000 Cases of Psoriasis* By L. DUNCAN BULKLEY, M.D. [Reprint.]

From H. K. LEWIS, London:

*Scab Healing.* By J. DELAPRATT HARRIS. 1893.

*From Hospital-Ward to Consulting-Room.* 1893.

*Public Health Laboratory Work.* By HENRY R. KENWOOD, M.B. 1893.

*Lewis's Diet Charts.*

*Diseases of Women.* By ARTHUR H. N. LEWERS, M.D. Fourth Edition. 1893.

*Alcohol and Public Health.* By J. JAMES RIDGE, M.D. Second Edition. 1893.

*Constitutional Syphilis.* By OSWALD ZIEMSSEN, M.D. 1893.

*Disinfectants and Antiseptics.* By EDWARD T. WILSON, M.B. Twenty-Fifth Thousand.

From LONGMANS, GREEN & CO., London:
*Abdominal Hernia.* By WILLIAM H. BENNETT. 1893.

From YOUNG J. PENTLAND, Edinburgh.
*Physical Diagnosis.* By G. A. GIBSON, M.D. AND WILLIAM RUSSELL, M.D. Second Edition. 1893.

From G. P. PUTNAM'S SONS, London:
*The Law of Psychic Phenomena.* By THOMSON JAY HUDSON. [1893.]

From F. J. REBMAN, London:
*When may Syphilitics Marry?* By Dr. SCHUSTER. Edited and Translated by Dr. C. RENNER. 1893.

From JOHN MORGAN RICHARDS, London:
*Medical Reprints.* March—May, 1893.

From HUNTER ROBB, M.D., Baltimore:

*Hysteromyomectomy for Large Myomata of the Uterus.* By HUNTER ROBB, M.D. [Reprint.]

From W. B. SAUNDERS, Philadelphia:
*A Text-Book of the Theory and Practice of Medicine.* By American Teachers. Edited by WILLIAM PEPPER, M.D. Vol. I. 1893.

From J. SHEPHERD CLARK CO., New York:
*La Revista Médico-Quirúrgica.* Febrero, Marzo. 1893.

From SIMPKIN, MARSHALL, HAMILTON, KENT & CO., LIMITED, London:
*Recreation.* By WILLIAM ODELL. 1893.

From J. W. SIMPSON, Derby:
*Twelfth Annual Report of the Derby Borough Asylum for* 1892.

From WINSTANLEY & WATKINS, Liverpool:
*Ambulance Card.* By LLEWELLYN MORGAN, M.D.

### Bristol Medico-Chirurgical Society.

---

### President.

E. MARKHAM SKERRITT, M.D., B.S., B.A. Lond.

### President-Elect.

J. GREIG SMITH, M.B., C.M., M.A. Aberd., F.R.S.E.

### Honorary Secretary.

G. MUNRO SMITH.

### Committee.

*Past Presidents.*

R. W. COE.
AUGUSTIN PRICHARD, M.D. Berlin.
J. G. SWAYNE, M.D. Lond.
E. LONG FOX, M.D. Oxon.
JAMES G. DAVEY, M.D. St. And.
W. JOHNSTONE FYFFE, M.D. Dub.
W. H. SPENCER, M.D., M.A. Cantab.
F. POOLE LANSDOWN.
L. M. GRIFFITHS.
R. SHINGLETON SMITH, M.D., B.Sc. Lond.
NELSON C. DOBSON.
S. H. SWAYNE.
F. R. CROSS, M.B. Lond.

J. MICHELL CLARKE, M.D., M.A. Cantab.
JOHN DACRE.
A. J. HARRISON, M.B. Lond.
W. H. HARSANT.
ARTHUR W. PRICHARD.
J. E. SHAW, M.B., C.M. Ed.

Medical Men wishing to join the Society are requested to communicate with the Honorary Secretary, G. MUNRO SMITH, 28 Alma Road, Clifton, Bristol. The Annual Subscription is 21/-.

---

Members of the Bristol Medico-Chirurgical Society, whose subscriptions to their Society are not more than twelve months in arrear, have free access to the Bristol Medical Library, University College.

---

*Extracts from Journal By-laws.*

4.—The *Journal* shall be delivered free to Subscribers and also to Members of the Society whose subscriptions are not in arrear.

6.—The Annual Subscription to the *Journal* for those who pay before the 1st of July shall be 5/-, post free, or 6/- if paid after that date.

---

Communications intended for insertion in the *Journal* should be sent to the Editor, Dr. SHINGLETON SMITH, Deepholm, Clifton Park, Clifton, Bristol.

Books for Review, Exchange Journals, and communications referring to the delivery of the *Journal* should be sent to the Assistant-Editor, L. M. GRIFFITHS, 9 Gordon Road, Clifton, Bristol, to whom Subscriptions are to be paid *in advance*.

Cases for Binding Vols. I.–X. are now ready, and may be obtained, price 7*d*. each (postage 2*d*. extra), upon application to J. W. ARROWSMITH, Quay Street, Bristol; or the *Journal* will be bound, including Case, for 1/3 per volume.

### Bristol Medico-Chirurgical Society.

---

### PAST PRESIDENTS.

1874—75. FREDERICK BRITTAN, M.D., B.A. Dub.

1875—76. ROBERT WILLIAM COE.

1876—77. SAMUEL MARTYN, M.D. Ed.

1877—78. AUGUSTIN PRICHARD, M.D. Berlin.

1878—79. HENRY EDWARD FRIPP, M.D. Würzburg.

1879—80. WILLIAM MICHELL CLARKE.

1880—81. JOSEPH GRIFFITHS SWAYNE, M.D. Lond.

1881—82. EDWARD LONG FOX, M.D. Oxon.

1882—83. JAMES GEORGE DAVEY, M.D. St. And.

1883—84. WILLIAM JOHNSTONE FYFFE, M.D. Dub

1884—85. GEORGE FORSTER BURDER, M.D. Aberd.

1885—86. WILLIAM HENRY SPENCER, M.D., M.A. Cantab.

1886—87. FRANCIS POOLE LANSDOWN.

1887—88. LEMUEL MATTHEWS GRIFFITHS.

1888—89. ROBERT SHINGLETON SMITH, M.D., B.Sc. Lond

1889—90. NELSON CONGREVE DOBSON.

1890—91. SAMUEL HENRY SWAYNE.

1891—92. FRANCIS RICHARDSON CROSS, M.B. Lond.

